# Role of the C-reactive protein-albumin ratio in predicting survival after breast cancer: A systematic review and meta-analysis

**DOI:** 10.12669/pjms.42.3.14536

**Published:** 2026-03

**Authors:** Hui Wang, Lu Yao

**Affiliations:** 1 Hui Wang, Department of Breast Surgery, Huzhou Central Hospital, Affiliated Central Hospital of HuZhou University, Huzhou, Zhejiang Province 313000, P.R. China; 2 Lu Yao, Department of Breast Surgery, Huzhou Central Hospital, Affiliated Central Hospital of HuZhou University, Huzhou, Zhejiang Province 313000, P.R. China

**Keywords:** C-reactive protein-albumin ratio, Breast cancer, Overall survival, Disease-free survival, Inflammation, Nutrition, Biomarker

## Abstract

**Objective:**

This review aimed to evaluate the association between C-reactive protein-albumin ratio (CAR) and survival outcomes in patients with breast cancer.

**Methodology:**

Databases of PubMed, Embase, Scopus, and Web of Science were searched up to 3^rd^ November 2025. Included studies involved adult patients with histologically confirmed breast cancer that reported hazard ratios (HRs) and 95% confidence intervals (CIs) for either overall survival (OS) or disease-free survival (DFS).

**Results:**

Six studies involving 2,427 patients were included. The pooled analysis demonstrated that elevated CAR was significantly linked with worse OS (HR = 2.66, 95% CI 1.64-4.31) and poorer DFS (HR = 2.39, 95% CI 1.50-3.82). Subgroup analyses revealed consistent associations across study design, cancer subtype, stage, country, and follow-up duration. Meta-regression showed that CAR cut-off value did not significantly influence the effect size for either OS or DFS. Sensitivity analyses indicated stable results.

**Conclusions:**

An elevated CAR may be associated with poorer OS and DFS in breast cancer. Given the scarce evidence, further research is needed to provide robust results.

***Registration No:*** PROSPERO database (CRD420251181780).

## INTRODUCTION

Breast cancer is the commonest and predominant cause of cancer deaths among women globally.^1^ Despite advances in surgery and targeted therapies, many patients still face recurrence or death.^2^,^3^ Traditional prognostic factors, like tumour size, grade, lymph node status, hormone receptors, and HER2, guide treatment.^4^ However, patients with similar profiles often have different outcomes, indicating other biological factors affect prognosis.^5^ This has increased interest in simple biomarkers that reflect tumour-host responses to improve risk assessment. Such biomarkers can be helpful especially for nursing personnel who are involved in pre-treatment preparation and post-treatment long-term care of breast cancer patients. Nurses can use simple biomarkers along with clinicians to easily prognosticate and provide adequate counselling to breast cancer patients.

Recent studies indicate that systemic inflammation is integral to the development, progression, and spread of tumours. Inflammatory cells and cytokines present in the tumour microenvironment can stimulate blood vessel formation, increase cell growth, and aid in metastasis.^6^ At the same time, the nutritional and immune health of the host significantly influences cancer advancement and the effectiveness of treatment.^7^ As a result, various indices based on inflammation and nutrition—such as the neutrophil-to-lymphocyte ratio (NLR), platelet-to-lymphocyte ratio (PLR), lymphocyte-to-monocyte ratio (LMR), prognostic nutritional index (PNI), and Glasgow prognostic score (GPS)—have been studied as potential prognostic markers across different types of cancers, including breast cancer.^8^,^9^

The C-reactive protein (CRP)-to-albumin ratio (CAR) has become a notable combined biomarker that reflects both inflammation and nutrition.^10^ Multiple studies have shown its prognostic significance across various cancers, like oral, biliary, hepatocellular, and colorectal, where higher CAR levels are linked to worse outcomes.^11^-^14^ However, evidence for breast cancer is still limited. Because of these findings, a thorough review of existing data is needed to better understand CAR’s prognostic value in breast cancer. This systematic review and meta-analysis thus aimed to quantify the relationship between CAR and survival metrics, such as overall survival (OS) and disease-free survival (DFS), in breast cancer patients.

## METHODOLOGY

We followed the PRISMA 2020 guidelines.^15^ The protocol was registered prospectively in the PROSPERO database (CRD420251181780).

### Eligibility criteria:

The PECOS framework guided the selection process for including or excluding studies. To be eligible, studies had to focus on adult women with histologically confirmed breast cancer and evaluate the prognostic significance of the CAR, which is obtained by dividing serum CRP levels by serum albumin levels. The comparison involved patients with high versus low CAR values, as determined by each study. Accepted study designs included observational analytical research, that reported survival outcomes, such as OS or DFS. These outcomes needed to be accompanied by hazard ratios (HRs) and 95% confidence intervals (CIs). Studies were excluded if they were reviews, case reports, conference abstracts, editorials, or laboratory animal experiments. Also, studies involving duplicate samples from the same cohort were omitted.

### Search strategy:

A thorough search was conducted across PubMed, Embase, Scopus, and Web of Science from inception to 3^rd^ November 2025, without language restrictions. Two reviewers (HW & LY) independently conducted the searches. The specific database search queries are detailed in Supplementary Table-I. Google Scholar was used to search the gray literature. References from included studies and reviews were checked by the reviewers for additional articles. All records were imported into EndNote (version 21) for organisation and deduplication. The screening process involved three steps. First duplicates were electronically removed. Second, both reviewers (HW & LY) screened the titles and abstracts of all remaining studies for initial eligibility and selected studies for full-text analysis. The reviewers then read the full texts of all downloaded articles to determine final inclusion. All disagreements were resolved through consensus.

**Supplementary Table-I:** Search strategy.

Database	Search strategy
PubMed	("Breast Neoplasms"[MeSH] OR "breast cancer"[Title/Abstract] OR "breast neoplasm"[Title/Abstract] OR "breast carcinoma"[Title/Abstract] OR "mammary carcinoma"[Title/Abstract]) AND ("C-Reactive Protein"[MeSH] OR "C reactive protein"[Title/Abstract] OR "C-reactive protein"[Title/Abstract] OR "CRP"[Title/Abstract]) AND ("Albumins"[MeSH] OR "albumin"[Title/Abstract]) AND ("ratio"[Title/Abstract] OR "CRP/Alb"[Title/Abstract] OR "CRP-albumin ratio"[Title/Abstract] OR "C-reactive protein albumin ratio"[Title/Abstract] OR "C-reactive protein-albumin ratio"[Title/Abstract]) AND ("survival"[Title/Abstract] OR "overall survival"[Title/Abstract] OR "OS"[Title/Abstract] OR "disease-free survival"[Title/Abstract] OR "DFS"[Title/Abstract] OR "progression-free survival"[Title/Abstract] OR "PFS"[Title/Abstract] OR "prognosis"[Title/Abstract] OR "mortality"[Title/Abstract] OR "death"[Title/Abstract])
Embase	("breast cancer"/exp OR "breast cancer":ab,ti OR "breast carcinoma":ab,ti OR "breast neoplasm":ab,ti) AND ("c reactive protein"/exp OR "c reactive protein":ab,ti OR "crp":ab,ti) AND ("albumin"/exp OR "albumin":ab,ti) AND ("crp/alb":ab,ti OR "crp alb":ab,ti OR "crp-albumin ratio":ab,ti OR "c-reactive protein albumin ratio":ab,ti OR "c reactive protein albumin ratio":ab,ti) AND ("survival":ab,ti OR "overall survival":ab,ti OR "os":ab,ti OR "disease free survival":ab,ti OR "dfs":ab,ti OR "prognosis":ab,ti OR "mortality":ab,ti)
Scopus	TITLE-ABS-KEY("breast cancer" OR "breast carcinoma" OR "breast neoplasm") AND TITLE-ABS-KEY("C-reactive protein" OR "CRP" OR "C reactive protein") AND TITLE-ABS-KEY("albumin") AND TITLE-ABS-KEY("CRP/Alb" OR "CRP-Alb" OR "CRP-albumin ratio" OR "C-reactive protein albumin ratio") AND TITLE-ABS-KEY("survival" OR "overall survival" OR "OS" OR "disease-free survival" OR "DFS" OR "prognosis" OR "mortality")
Web of Science	TS=("breast cancer" OR "breast carcinoma" OR "breast neoplasm") AND TS=("C-reactive protein" OR "CRP" OR "C reactive protein") AND TS=("albumin") AND TS=("CRP/Alb" OR "CRP-Alb" OR "CRP-albumin ratio" OR "C-reactive protein albumin ratio") AND TS=("survival" OR "overall survival" OR "OS" OR "disease-free survival" OR "DFS" OR "prognosis" OR "mortality")

### Data extraction:

Two reviewers (HW & LY) separately extracted data using a standardised collection form. They recorded information such as the first author’s name, publication year, country, study design, sample size, and demographic and clinical details like mean or median age, tumour stage, treatment options, and follow-up duration. Additional details included the timing of CAR measurement, the cut-off values used, and the method for establishing these thresholds. The main outcomes were OS and DFS. When both univariate and multivariate analysis results were available, data from multivariate models were preferred. Any disagreements among reviewers were settled by reaching a consensus, and the authors of the original studies were contacted to obtain any missing critical data.

### Quality assessment:

The risk of bias in studies was appraised utilising the Newcastle-Ottawa Scale (NOS) tailored for cohort research.^16^ This assessment tool examines three fundamental components: the process of selecting study participants, the comparability of cohorts through design or analytical approaches, and the verification of outcomes. Two independent reviewers evaluated each study, and any discrepancies were resolved through discussions. Based on the aggregate scores, studies were classified into three categories: high quality (scores 7-9), moderate quality (scores 4-6), or low quality (scores three or below).

### Statistical analysis:

The analyses were done in R (version 4.3) using the “meta” and “metafor” packages. HRs with 95% CIs were either extracted or estimated to evaluate the link between elevated CAR and survival outcomes in breast cancer. A random-effects model based on the DerSimonian-Laird method was used to compute pooled estimates. Heterogeneity was assessed with Cochran’s Q test and quantified using the I² statistic. I^2^ values of >50% indicated high heterogeneity. Subgroup analyses explored potential sources of heterogeneity, including country, study design, type of cancer, stage, and follow-up duration. Meta-regression was also performed to examine the influence of the CAR cut-off on pooled outcomes. Sensitivity analysis was performed by sequentially excluding one study at a time to evaluate the stability of the results. Publication bias was examined by visual inspection of funnel plot asymmetry. As the number of included studies was <10, formal statistical tests for funnel plot asymmetry (Egger’s or Begg’s tests) were not performed, and interpretation was based on visual assessment alone.

## RESULTS

### Search results:

**Fig.1:**
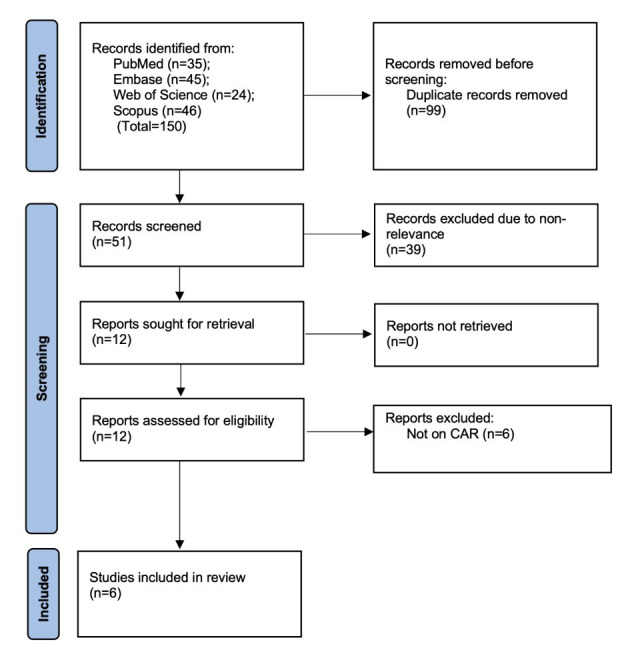
PRISMA flowchart.

The search across PubMed, Embase, Scopus, and Web of Science found 150 records. After removing 99 duplicates, 51 studies remained. Screening titles and abstracts excluded 39 articles, leaving 12 for full-text review. Six were excluded because they did not examine CAR in relation to breast cancer outcomes. Ultimately, six studies^17^-^22^ met the inclusion criteria (Fig.1).

### Study characteristics:

The pooled sample size of the studies was 2,427 patients (Table-I). Most studies were conducted in China, while one was from Indonesia. The study designs were primarily retrospective, with one prospective cohort. Patient age across studies ranged from approximately 45 to 57 years. Most studies included all types of breast cancer, while two studies were only on luminal subtype. Treatments received by participants included surgery, chemotherapy, radiotherapy, and endocrine therapy, either alone or in combination. The CAR cut-off values applied varied substantially among studies, ranging from 0.029 to 1.5. The reported follow-up durations spanned from approximately 45 to 62 months. Quality assessment indicated that all included studies were of high methodological quality, with total scores ranging from 7 to 9.

**Table-I:** Details of included studies.

Study	Design	Type of breast cancer	Sample size	Age (years)	Stage	Treatment	CAR cut-off	Outcomes, HR (CI)	Follow-up (m)	NOS score
Hutajulu 2025 Indonesia	R	All types	202	51 [45.4-57.6]	I-IV	First line chemotherapy	1.5	OS: 2.16 (1.27, 3.68) DFS: 2.10 (1.10, 3.99)	46 (1-77)	S-4 C-2 O-3
Ruan 2023 China	P	All types	514	53.7± 10.3	I-IV	Surgery, radiotherapy, chemotherapy	0.24	OS: 2.56 (1.46, 4.47)	43.1 (40.7-49.6)	S-4 C-2 O-3
Chen 2022 China	R	Luminal breast cancer with HER-ve	708	52.4 (15-95)	I-III	Surgery, radiotherapy, chemotherapy	0.07	OS: 6.56 (3.558, 12.106) DFS: 3.858 (2.346, 6.345)	62.7 (12-91)	S-4 C-2 O-3
Liu 2021 China	R	Luminal B subtype breast cancer	199	48 (25-72)	I-III	Surgery and endocrine therapy	0.44	OS: 1.874 (1.226, 2.864) DFS: 4.346 (1.477, 12.786)	NR	S-4 C-2 O-2
Wang 2020 China	R	With skeletal metastases	212	55	I-IV	Surgery, radiotherapy, chemotherapy	0.34	OS: 1.34 (0.817, 2.198) DFS: 1.297 (0.791, 2.127)	45	S-4 C-2 O-3
Zhou 2019 China	R	Non-metastatic breast cancer	592	51 (27-77)	I-III	Surgery, radiotherapy, chemotherapy	0.029	OS: 9.189 (2.079, 40.621) DFS: 2.225 (1.113, 4.451)	60 (10-120)	S-4 C-2 O-3

NR, not reported; P, prospective; R, retrospective; NOS, Newcastle Ottawa scale; m, months; CAR, C-reactive protein albumin ratio; OS, overall survival; DFS, disease free survival; Selection of cohort; C, comparability; O, outcome assessment; HR, hazard ratio; CI, confidence intervals.

### Meta-analysis:

**Fig.2:**
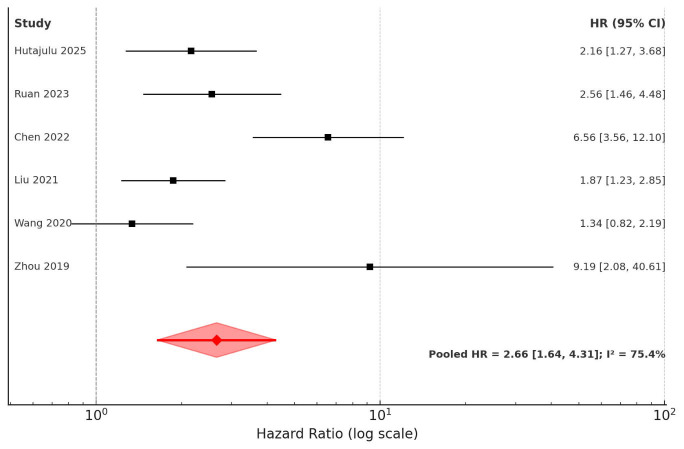
Forest plot for OS in breast cancer patients based on CAR.

**Supplementary Fig.1:**
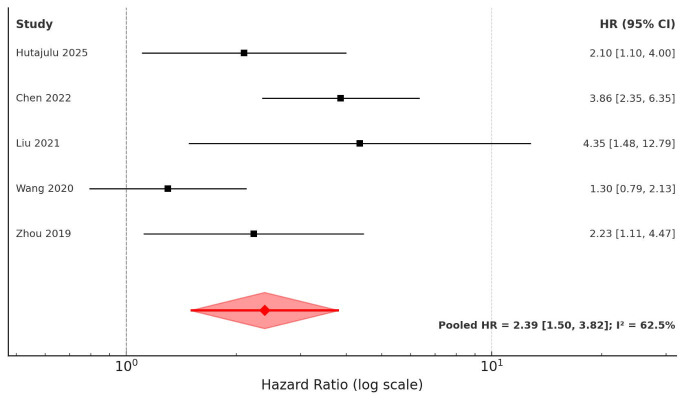
Funnel plot for overall survival.

The pooled results from the random-effects meta-analysis showed that higher CAR was significantly associated with poorer OS in breast cancer patients (HR: 2.66, 95% CI: 1.64-4.31; p < 0.001) (Fig.2). There was notable heterogeneity among the studies (I² = 75.4%). Sensitivity analyses indicated that removing any single study did not alter the significance of the effect size (Supplementary Table-II). The funnel plot showed slight asymmetry, suggesting the possibility of small-study effects (Supplementary Fig.1). The association’s direction was consistent across subgroup analyses based on country, study design, breast cancer type, disease stage, and follow-up period (Table-II). On meta-regression analysis, the CAR cut-off value did not significantly influence the effect size for OS (Beta: -0.48, standard error: 0.56, p=0.43).

**Supplementary Table-II:** Results of sensitivity analysis.

OS			
Excluded study	HR	-95% CI	+95% CI
Hutajulu 2025	2.850101	1.555469	5.222269
Ruan 2023	2.737975	1.505791	4.978452
Chen 2022	2.063453	1.450079	2.936279
Liu 2021	2.970277	1.600662	5.511812
Wang 2020	3.104171	1.84714	5.216648
Zhou 2019	2.404787	1.493143	3.873037
DFS			
*Excluded study*	*HR*	*-95% CI*	*+95% CI*
Hutajulu 2025	2.50194	1.368989	4.572502
Chen 2022	1.973391	1.282602	3.036228
Liu 2021	2.204176	1.33065	3.651142
Wang 2020	2.939197	2.075044	4.163227
Zhou 2019	2.458743	1.358034	4.451593

CI, confidence intervals; HR, hazard ratio.

**Table-II:** Results of subgroup analysis.

Moderator	Group	Studies	HR [95% CI]	I² (%)
OS				
Country	China	5	2.85 [1.56-5.22]	80.2
	Indonesia	1	2.16 [1.27-3.68]	100
Design	Prospective	1	2.56 [1.46-4.48]	100
	Retrospective	5	2.74 [1.51-4.98]	80.2
Type of Breast Cancer	All types	4	2.23 [1.35-3.70]	59.5
	Luminal	2	3.43 [1.00-11.73]	90.9
Stage	I-III	3	4.30 [1.50-12.38]	84.9
	I-IV	3	1.92 [1.30-2.82]	38.2
Follow-up	>5 years	2	6.89 [3.91-12.13]	0.0
	<5 years	3	1.92 [1.30-2.82]	38.2
DFS				
Country	China	4	2.50 [1.37-4.57]	71.6
	Indonesia	1	2.10 [1.10-4.00]	0
Type of Breast Cancer	All types	3	1.70 [1.20-2.43]	5.8
	Luminal	2	3.94 [2.51-6.19]	0
Stage	I-III	3	3.33 [2.28-4.86]	0
	I-IV	2	1.58 [0.99-2.50]	25.2
Follow-up	<5 years	2	1.58 [0.99-2.50]	25.2
	>5 years	2	3.10 [1.83-5.25]	37

CI, confidence intervals; HR, hazard ratio; OS, Overall survival; DFS, disease free survival.

**Fig.3:**
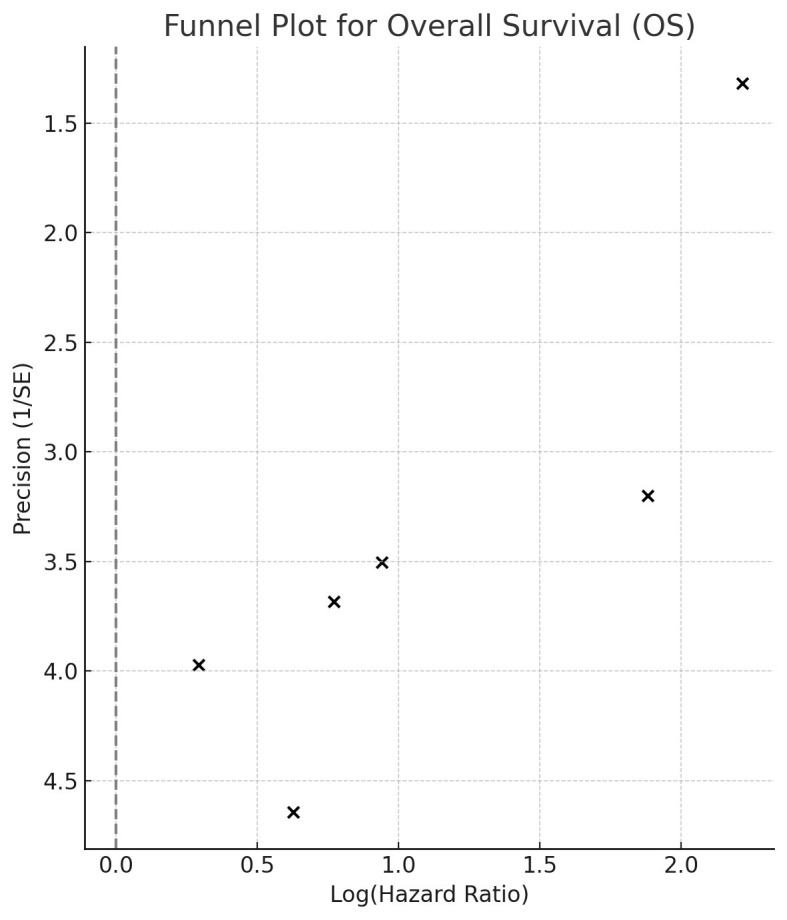
Forest plot for DFS in breast cancer patients based on CAR.

**Supplementary Fig.2:**
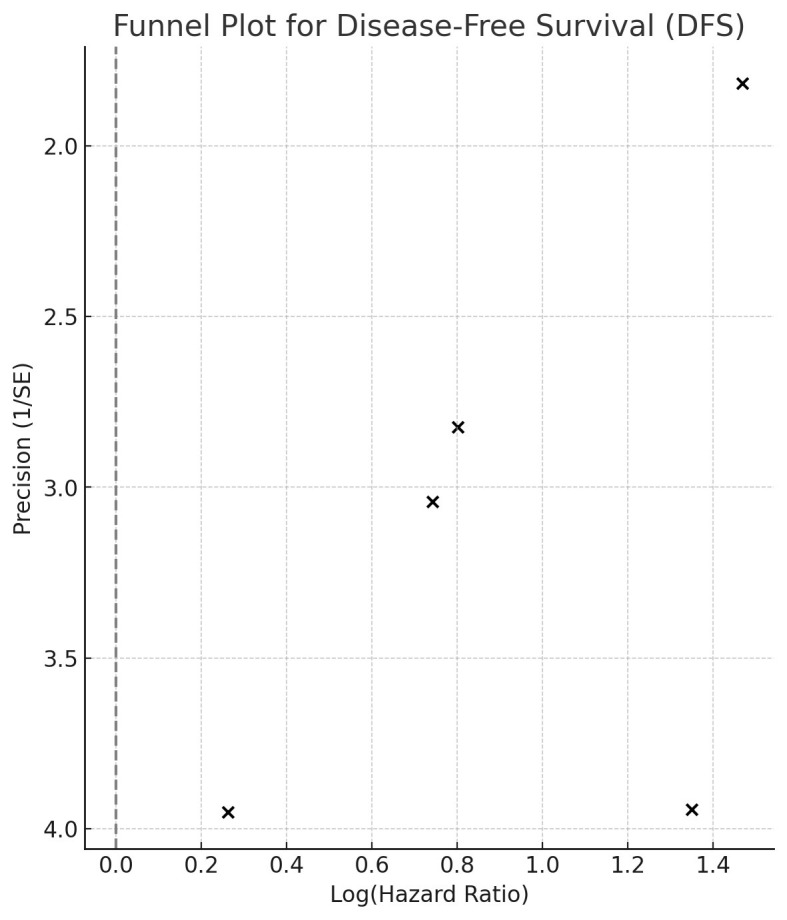
Funnel plot for.

The combined analysis of five studies indicated that patients with elevated CAR levels had significantly worse DFS (HR: 2.39, 95% CI: 1.50-3.82, I² = 62.5%) (Fig.3). Sensitivity analysis confirmed the stability of the association (Supplementary Table-I). Subgroup analyses across different countries, study designs, cancer types, disease stages, and follow-up times showed consistent results (Table-II). Inspection of the funnel plot showed mild asymmetry but no strong evidence of publication bias (Supplementary Fig.2). In the meta-regression, the CAR cut-off value did not significantly moderate the prognostic association between CAR and DFS in breast cancer (Beta: -0.17, standard error: 0.46, p=0.73).

## DISCUSSION

This meta-analysis, comprising six studies with 2,427 patients diagnosed with breast cancer, demonstrated that an elevated CAR is significantly correlated with poorer OS and DFS. The pooled analysis indicated that higher CAR values are associated with an increased mortality risk, with a combined HR of 2.66, signifying more than a twofold greater risk of death among patients exhibiting elevated CAR levels. Likewise, in terms of DFS, the pooled HR was 2.39, suggesting that higher CAR levels also increase the likelihood of disease recurrence or progression.

The results of this meta-analysis align with earlier systematic reviews that examined the prognostic significance of the CAR across different cancers. A recent study by Utsumi et al^14^ analysed preoperative CAR in patients with biliary tract cancer and found that higher CAR levels were significantly associated with worse postoperative survival. Similarly, Lu et al.’s^12^ review of oral squamous cell carcinoma indicated that elevated CAR was strongly associated with poorer OS. In metastatic colorectal cancer, a meta-analysis by Pan et al,^13^ including six studies and 771 patients, showed that increased CAR was significantly associated with reduced OS and DFS. Additionally, Lin et al’^11^ meta-analysis involving hepatocellular carcinoma patients revealed that a higher CAR not only predicted worse survival outcomes but was also associated with more aggressive clinicopathological features, such as larger tumours, vascular invasion, and advanced stages.

There was substantial heterogeneity among the included studies, especially regarding OS (I² = 75.4%) and, to a lesser extent, DFS (I² = 62.5%). This variation likely results from differences in patient characteristics, tumour stage, treatment strategies, and methodological factors, such as CAR cut-off points. Subgroup analyses helped clarify some of these sources of variation. The association between elevated CAR and poor survival outcomes was consistent across all subgroups, demonstrating that the prognostic significance of CAR is robust. Nonetheless, there was no reduction in the inter-study heterogeneity for the analysis on OS, indicating that other unknown factors may be at play. Given the limited data from the included studies, this review couldn’t separate data by specific cancer stage or treatment protocols, which may have provided stronger clinical evidence.

A major source of variability was the differing cut-off points for CAR used across various studies. To explore this further, a meta-regression was performed. The slope of the regression was negative but not statistically significant, indicating that changes in the threshold for high CAR did not strongly influence its association with outcomes. The variation in CAR cut-off values among studies poses a significant challenge for clinical implementation. In this review, thresholds to distinguish high from low CAR ranged broadly from 0.029 to 1.5. This inconsistency hinders comparability between studies and complicates the establishment of a universal clinical reference range. Practically, this suggests that CAR should currently be viewed as a relative, not absolute, marker of inflammatory and nutritional status. Elevated CAR values reliably identify patients at higher risk of recurrence or death, but the specific “high risk” threshold may vary by institution, disease stage, and regional populations. Future research should focus on developing and validating standardized CAR cut-offs through large, multi-centre cohorts and receiver operating characteristic analyses to promote consistency and reproducibility.

The biological basis linking the CAR to cancer prognosis centres on systemic inflammation and nutritional health, both of which are critical to tumour development.^11^,^13^ CRP, an acute-phase protein synthesised by the liver in response to inflammatory cytokines like interleukin-6 and tumour necrosis factor-alpha, acts as a marker for tumour-related inflammation.^23^,^24^ High CRP levels support a tumour-friendly environment by promoting blood vessel growth, reducing cell death, and increasing the proliferation and spread of cancer cells.^25^ Additionally, elevated CRP can boost estrogen production and influence hormone receptor activity, which can affect tumour aggressiveness and metastasis, especially in breast cancer.^19^

Conversely, serum albumin is widely recognised as a key marker of nutritional and physiological reserves. Hypoalbuminemia indicates malnutrition and systemic inflammation, as chronic inflammation suppresses liver albumin production and accelerates its breakdown.^26^ Low albumin levels weaken immune defences, diminish antioxidant capacity, and impair drug metabolism and treatment tolerance, leading to worse clinical outcomes.^27^ Therefore, CAR serves as an integrated marker reflecting both inflammatory response and nutritional decline. A higher CAR indicates increased inflammation and protein-energy deficiency, conditions that promote tumour growth, immune escape, and resistance to therapy.^21^

### Limitations:

Although this represents the first systematic review on the topic, there are several limitations to consider. Firstly, the number of included studies was relatively limited, primarily involving Asian populations, which may limit the applicability of the results to other ethnic or geographic groups. Secondly, all the studies were observational, mainly retrospective, which makes them prone to selection bias, residual confounding, and incomplete adjustment for clinical variables. Thirdly, there was significant heterogeneity, likely due to differences in patient characteristics, disease stages, treatment approaches, and, notably, the wide range of CAR cut-off values used to define high-risk patients. This variability underscores the lack of a standardised threshold for CAR, potentially undermining the reproducibility of findings across settings. Studies also did not clearly specify the metastatic status of the patients which precluded a subgroup analysis on this important variable. Fourth, most studies used a single pre-treatment CAR measurement without accounting for changes over time during therapy, which might yield more valuable prognostic insights. Lastly, the risk of publication bias cannot be entirely ruled out, as studies with null or non-significant results may not have been published.

The review carries important clinical implications. The consistent results across various study settings, despite methodological differences, emphasise CAR as a valuable and accessible biomarker that can enhance traditional risk assessment methods. The marker is a straightforward and cost-effective method that nursing personnel can employ to identify breast cancer patients who are at a higher risk of a poor prognosis as a result of underlying inflammation or malnutrition. Nurses can collaborate with the multidisciplinary team to instigate early nutritional interventions, optimize supportive care, and enhance treatment tolerance by monitoring CAR values. This proactive strategy may allow for tailored patient management and better overall outcomes.

Future research in the form of large, multicenter studies is needed to confirm the prognostic value of the CAR ratio across diverse populations, given that most current evidence stems from Asian cohorts. Establishing standardised CAR cut-off values using harmonised statistical methods, such as receiver operating characteristic–based optimisation and external validation, is essential for ensuring consistency and clinical applicability. Studies should also investigate how CAR changes over time during treatment, rather than relying solely on a pre-treatment measurement, as tracking its longitudinal variation may better indicate treatment response and inflammatory status. Combining CAR with clinicopathological factors and molecular subtypes could lead to more accurate risk models. Finally, interventional trials examining whether targeted nutritional or anti-inflammatory interventions can alter CAR and improve patient survival would offer valuable insights into its potential as a modifiable prognostic biomarker.

## CONCLUSION

Our review suggests that there may be an association between elevated CAR and poor OS and DFS in patients with breast cancer. Further prospective multicentric studies with larger sample size are needed to validate the present results.

### Authors’ contributions:

**HW:** Literature search, study design and manuscript writing.

**HW and LY:** Data collection, data analysis and interpretation. Critical Review.

**HW:** Was involved in the manuscript revision and validation and is responsible for the integrity of the study. All authors have read and approved the final manuscript.
